# Optimizing base editors for improved efficiency and expanded editing scope in rice

**DOI:** 10.1111/pbi.13124

**Published:** 2019-05-18

**Authors:** Mugui Wang, Zhidan Wang, Yanfei Mao, Yuming Lu, Ruifang Yang, Xiaoping Tao, Jian‐Kang Zhu

**Affiliations:** ^1^ Shanghai Center for Plant Stress Biology and Center for Excellence in Molecular Plant Sciences Chinese Academy of Sciences Shanghai China; ^2^ University of Chinese Academy of Sciences Beijing China; ^3^ Crop Breeding and Cultivation Research Institute Shanghai Academy of Agricultural Sciences Shanghai China; ^4^ Department of Horticulture and Landscape Architecture Purdue University West Lafayette IN USA

**Keywords:** gene editing, base editing, biotechnology, rice, crop improvement, cytidine deaminase, adenine deaminase

Dear Editor,

Base editors, presently including cytidine base editors (CBEs) and adenine base editors (ABEs), enable precise base alterations in the genome without inducing DNA double‐stranded breaks (DSBs). Base editors are valuable tools for precision plant molecular breeding since many agronomic traits are controlled by variations in one or few DNA bases. The early developed CBE and ABE systems, consisting of the rat cytidine deaminase APOBEC1 (rAPOBEC1) or activation‐induced cytidine deaminase (AID) PmCDA1, and the evolved tRNA adenine deaminase TadA, respectively, have been applied to many plant species. To improve the base editing efficiency, more effective cytidine deaminases such as the human APOBEC3A have been tested (Zong *et al*., 2018). On the other hand, for expanding the base editing scope in plants, several SpCas9 and SaCas9 variants such as VQR‐Cas9, VRER‐Cas9 and SaKKH‐Cas9 that recognize PAMs other than the canonical NGG motif were introduced into the CBE and ABE toolbox (Hua *et al*., 2018a; Qin *et al*., 2018). However, relative to the widely used CRISPR/Cas gene editing technologies for inducing DSBs and subsequent repair‐caused mutations, the efficiency of base editing is still low. In addition, base editors reported thus far are constrained by recognition of only a few kinds of PAM sequences.

We have previously reported the initial adoption of CBEs and ABEs in rice (Hua *et al*., 2018b; Lu and Zhu, 2017). In our CBE system, we fused rAPOBEC1 to the N‐terminus of SpCas9 nickase (Cas9n, D10A) using the unstructured 16‐residue peptide XTEN as linker. A traditional nuclear localization signal, SV40 NLS peptide, was added to the C‐terminus of the Cas9n. Two agronomically important genes of rice, *NRT1.1B* and *SLR1*, were selected for editing by this CBE system. However, the base substitution efficiencies were low, with only 2.7% for *NRT1.1B* and 13.3% for *SLR1*, respectively (Lu and Zhu, 2017). In our ABE system, we synthesized wild‐type ecTadA and its mutant form ecTadA*7.10 and linked them together using a 32‐amino acid (aa) linker; the resulting recombinant protein was fused to the N‐terminus of the SpCas9 or SaCas9 nickase with the same linker. Testing at different targets showed that the base substitution efficiencies ranged from 5% to 60%, with most of the target sites having efficiencies lower than 30% as reported by other groups (Hua *et al*., 2018b).

Recently, Koblan *et al*. (2018) found that the expression levels of base editors are major bottlenecks for base editing efficiency. They improved BE4 and ABE7.10 base editors by adopting bipartite nuclear localization signals (bpNLS), optimizing codon usage and ancestral reconstruction of the deaminase component. The resulting BE4max, AncBE4max and ABEmax editors showed increased editing efficiencies in a variety of settings, especially under suboptimal conditions or at sites previously edited with low efficiencies (Koblan *et al*., 2018). To improve the base editing efficiency in plants, we directly adopted the above GenScript codon‐optimized nucleotide sequences of bpNLS‐Anc689 APOBEC‐32 aa Linker and bpNLS‐adenine deaminase of ABE7.10‐32 aa Linker into our previous CBE and ABE editors, resulting in Anc689BE4max and ABEmax, respectively (Figure 1a,b).

**Figure 1:**
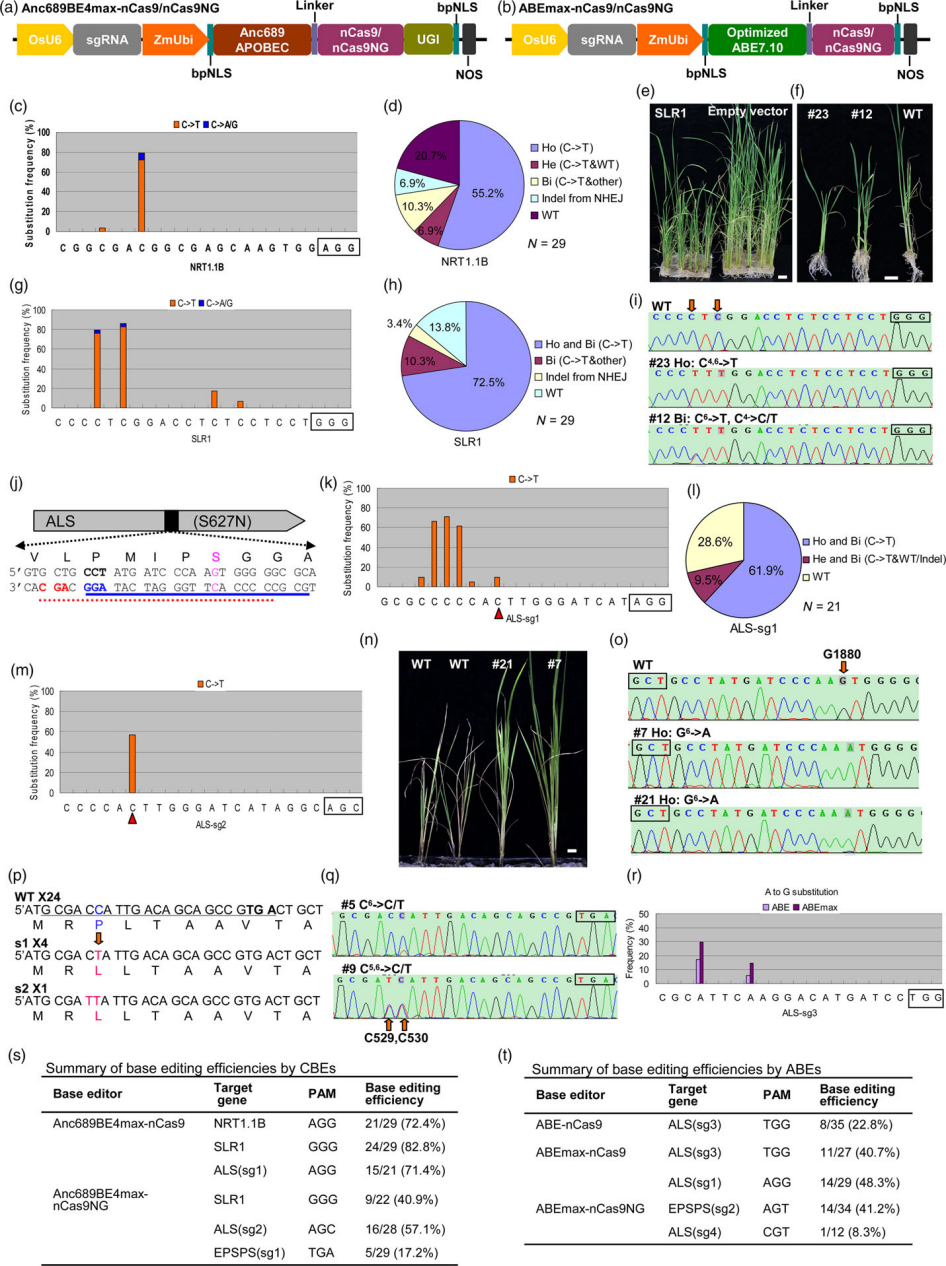
Optimizing base editors for improved efficiency and expanded editing scope in rice. (a and b) Constructs of the Anc689BE4max (a) and ABEmax (b) base editors. Optimized ABE7.10 refers to the GenScript codon‐optimized sequence of adenine deaminase of ABE7.10; this sequence and Anc689APOBEC were directly derived from Koblan *et al*. (2018). (c,g) Frequencies of base substitutions at the target sites of *NRT1.1B* (c) and *SLR1* (g); the PAM motif is marked in box. (d,h,l) Distribution of the genotypes from transgenic rice plantlets edited at the *NRT1.1B* (d), *SLR1* (h) and *ALS* (l) target sites. Ho: homozygous, Bi: biallelic, He: heterozygous, WT: wild type, Other: other base substitutions but not C‐>T. N: the total number of identified plantlets. (e,f) Phenotype of the regenerated rice plantlets from base editing at *SLR1*. Scale bar equal to 1 cm. (i,o,q) Representative Sanger sequencing chromatograms at the *SLR1* (i), *ALS* (o) and *EPSPS* (q) target sites. The plant ID (#), genotype and its base substitution status are shown above each chromatogram. The superscript indicates the base position within protospacer. The substituted bases are also marked by red arrows, and their positions in the gene are indicated in number. The PAM motif is marked in box. Ho: homozygous, Bi: biallelic, He: heterozygous, WT: wild type. (j) The target sites designed for base editing at the ALS^S627N^ of rice. The sgRNA‐PAM sequences designed for Anc689BE4max‐nCas9 and Anc689BE4max‐nCas9NG are underlined in blue and red, respectively, and the PAM motif is marked in bold. The intended base and amino acid for substitution are marked in pink. (k,m) Frequencies of base substitutions at the target sites of ALS‐sg1 (k) and ALS‐sg2 (m). The PAM motif is marked in box, and the red triangles indicate the intended base for conversion. (n) Phenotype of the transgenic rice plantlets treated by herbicide. 0.03% Imazethapyr (Shandong CYNDA) was sprayed on the plantlets, and the photograph was taken 25 days after treatment. Scale bar equal to 1 cm. (p) Wild type and the mutated sequences of *EPSPS*. The designed sgRNA‐PAM sequences are underlined, and the PAM motif is further marked in bold. The intended base and amino acid before and after editing are marked in blue and pink, respectively. The quantity of each genotype from transgenic plantlets is indicated by ×. s1, single nucleotide substitution mutation; s2, two nucleotides substitution mutation, WT, wild type. (r) Frequencies of base substitutions at the target site of ALS‐sg3 edited by ABE and ABEmax. The PAM motif is marked in box. (s,t) Summary of editing efficiencies for different base editors. Base editing efficiency was calculated by scoring the number of plantlets with anticipated base substitution within the target site relative to the total number of identified transgenic plantlets. The designed sgRNA‐PAM sequences for EPSPS‐sg2 and ALS‐sg4 are 5′‐GAGAAGGATGCGAAAGAGGAAGT and 5′‐TAACAAAGAAGAGTGAAGTCCGT, respectively.

To directly compare the performance of Anc689BE4max with our previous CBE, the *NRT1.1B* and *SLR1* were selected for editing using the previously tested sgRNA. As shown in Figure 1c, 72.4% of the transgenic rice lines harboured the target C to T replacement at *NRT1.1B* target site, and 76.2% of these lines (55.2% of total transgenic lines) are homozygous (Figure 1d). Most of the regenerated plantlets transformed with Anc689BE4max‐sgRNA^SLR1^ displayed an obvious dwarf phenotype (Figure 1e,f). Genotyping and sequencing results showed that 82.8% of the transgenic lines converted C to T at their target site, and 72.5% of the transgenic lines were homozygous or biallelic C to T substitutions (Figure 1g,h). The sequencing results are also consistent with the phenotype of each plantlet (Figure 1f,i). Compared with the efficiency of 2.7% for *NRT1.1B* and 13.3% for *SLR1* from our previous CBE (Lu and Zhu, 2017), the Anc689BE4max showed much higher base editing efficiencies. We also noticed that the deamination window ranged from the 4th to 15th target bases, but the substitutions were concentrated at the 4th to 7th bases, which is similar to our previously reported CBE (Lu and Zhu, 2017).

To further evaluate the efficiency of Anc689BE4max, we designed an sgRNA (ALS‐sg1) for modifying the acetolactate synthase gene (*ALS*) in rice. It is known that a mutated form of ALS, ALS^S627N^ (G^1880^ to A in Nipponbare DNA sequence), confers tolerance to imidazolinone herbicides (Piao *et al*., 2018) (Figure 1j). Similar to the results from the base editing of *NRT1.1B* and *SLR1*, 71.4% of the transgenic lines contained C to T substitution at their target site in *ALS*, and most of them were homozygous or biallelic (Figure 1k,l). Our result also shows that although the target C ranging from 4th to 10th of the protospacer could be replaced by T, the substitution preferentially occurred within the window from the 5th to 7th base. Only two of the edited lines contained the intended G^1880^ to A conversion, since this target base is located outside of the ‘hot spot’ of the deamination window (Figure 1k).

Recently, Nishimasu *et al*. (2018) reported that a rationally engineered SpCas9 variant, SpCas9‐NG, containing the R1335A/L1111R/D1135V/G1218R/E1219F/A1322R/T1337R seven amino acid alteration, can recognize relaxed NG PAMs in human cells. During the preparation of our manuscript, Endo *et al*. (2018) reported that the nickase of this variant (D10A) fused to cytidine deaminases such as PmCDA1 and rAPOBEC1 could mediate C to T conversion at sites bearing NG PAMs in rice calli, but the nSpCas9NG‐APOBEC1 base editor showed a low activity at most of the tested target sites. To expand the access range of base editors, we adopted the SpCas9NG nickase into our Anc689BE4max and ABEmax to replace the SpCas9 nickase, resulting in Anc689BE4max‐nCas9NG and ABEmax‐nCas9NG system, respectively (Figure 1a,b). Testing of the Anc689BE4max‐nCas9NG system at *SLR1* using the same sgRNA with GGG PAM showed an editing efficiency of 40.9%, lower than that of Anc689BE4max‐nCas9 (Figure 1s).

To facilitate the G^1880^ to A substitution in *ALS*, we designed another sgRNA, ALS‐sg2, harbouring the AGC PAM, for editing by the Anc689BE4max‐nCas9NG system (Figure 1j). 57.1% of the transgenic lines showed the intended G^1880^ to A replacement, although the combined base substitution efficiency was lower than that of Anc689BE4max‐nCas9 with ALS‐sg1 (Figure 1m,s). The mutants homozygous for the G^1880^ to A substitution were tolerant to imidazolinone herbicide, whereas wild‐type plants were not (Figure 1n,o). Taking advantage of the nCas9NG‐derived base editor that can recognize relaxed PAMs, we further applied it to modify the 5‐enolpyruvylshikimate‐3‐phosphate synthase (*EPSPS*) gene. Previous work showed that a single base transition of C^317^‐T within *OsEPSPS* (C^530^ in Nipponbare genome), changing proline‐106 to leucine (P106L), led to resistance to another herbicide, glyphosate, in the EPSPS‐deficient *Escherichia coli* strain AB2829 (Zhou *et al*., 2006). Here, we designed an sgRNA harbouring the TGA PAM (EPSPS‐sg1) for editing by our Anc689BE4max‐nCas9NG system, since there are no suitable NGG PAMs near the protospacer (Figure 1p). We successfully obtained five plantlets containing the targeted C^530^ to T replacement from 29 transgenic lines (Figure 1p,q,s). The glyphosate tolerance will be tested in the T1 generation since there was no homozygous mutant in the T0 plants.

To evaluate the activity of our ABEmax editor, we designed a third sgRNA (ALS‐sg3) to edit the *ALS* gene by ABEmax and our previous ABE side by side. The results showed that ABEmax doubled the editing efficiency of ABE (Figure 1r,t). We further tested ABEmax at the ALS‐sg1 target site, and the results showed that 48.3% of the transgenic lines harboured A to G substitution (Figure 1t). The general editing efficiencies of ABEmax seem lower than those of Anc689BE4max (Figure 1s,t). We further evaluated the ABEmax‐nCas9NG system with non‐canonical NGG PAMs. Testing at the EPSPS‐sg2 target site harbouring the AGT PAM showed an editing efficiency of 41.2%. However, testing at another target site (ALS‐sg4) bearing the CGT PAM showed the editing efficiency lower than 10% (Figure 1t).

In summary, our upgraded base editors not only show substantially increased editing efficiencies, but also have expanded editing scopes compared to previously reported CBEs and ABEs. These improved base editors are more powerful tools for molecular breeding of crops, although more plant species and more target sites need to be tested in the future.

## Conflict of interest

The authors declare no conflicts of interest with respect to this work.
